# Susceptible-Infected-Susceptible Epidemic Discrete Dynamic System Based on Tsallis Entropy

**DOI:** 10.3390/e22070769

**Published:** 2020-07-14

**Authors:** Shaher Momani, Rabha W. Ibrahim, Samir B. Hadid

**Affiliations:** 1 Department of Mathematics and Sciences, College of Humanities and Sciences, Ajman University, Ajman 346, UAE; shahermm@yahoo.com (S.M.); s.hadid@ajman.ac.ae (S.B.H.); 2 Department of Mathematics, Faculty of Science, University of Jordan, Amman 11942, Jordan; 3 Informetrics Research Group, Ton Duc Thang University, Ho Chi Minh City 758307, Vietnam; 4 Faculty of Mathematics & Statistics, Ton Duc Thang University, Ho Chi Minh City 758307, Vietnam

**Keywords:** Tsallis entropy, discrete dynamic system, equilibrium point, COVID-19, fractional calculus

## Abstract

This investigation deals with a discrete dynamic system of susceptible-infected-susceptible epidemic (SISE) using the Tsallis entropy. We investigate the positive and maximal solutions of the system. Stability and equilibrium are studied. Moreover, based on the Tsallis entropy, we shall formulate a new design for the basic reproductive ratio. Finally, we apply the results on live data regarding COVID-19.

## 1. Introduction

Discrete dynamic systems of SISE were extensively discussed for a long historical period, that successfully described the procedure in disease diffusion (see [1]). A decay ago, in the year 1927, the traditional SISE was offered [2]. After that, there established an enormous number of periodicals on SISE [3,4,5]. In overall, SISEs are considered to be homogeneously combined, which indicates that susceptible persons are infected with the same information. Nevertheless, there are various systems of populations in human culture [6], and the joining between persons is not identical. The stability and convergence of the systems are studied by using the basic reproductive ratio. This ratio is given in different formula based on the system and the situation of the solution. In our discussion, we suggest new formal of this ratio based on the entropy concept. For COVID-19, the researchers established a suitable ratio called the case fatality rate (CFR).

In [7], the researchers studied SISE at level-liberated networks; it designates that under the suitable parameters, there is probably a threshold at which the disease will persevere. In view of [7], some inoculation approaches are investigated, which additional develop the mechanisms of SISE on networks [8,9,10]. Obviously, the classes of difference equations have numerous practices in SISE system [11,12,13]. In reality, a positive interval, and discrete simulations often give information about disease [14]. On the other hand, a difference equation is the discretion of the continuous model [15,16], which indicates it practical to respond to the approximation method. Particularly, discrete simulations show a more complex dynamical conduct than the conforming continuous representations [17,18,19,20].

Under these compensations in attention, the state of the discrete SISE system of networks is about excessive investigation care. From the above-mentioned details, we shall deal with a discrete-time SISE system involving Tsallis entropy, which will be important work. We apply the results to live data regarding COVID-19.

## 2. The SISE Dynamical System Involves Tsallis Entropy

In traditional statistical methods, the entropy function formerly presented by Rudolf Clausius is construed as statistical entropy utilizing probability theory. The statistical entropy view was introduced in 19th century with the work of physicist Ludwig Boltzmann. This entropy was generalized by Tsallis as follows [21]: Consider a discrete set of probabilities $\left\{\rho_{j}\right\}$ satisfying the condition $\sum_{j}\rho_{j}=1$, and α any real number, the Tsallis entropy is formulated by the terms

$$\Upsilon_{\alpha }\left(\rho_{j}\right)=\frac{1}{\alpha -1}\left(1-\sum_{j}^{n}\rho_{j}^{\alpha }\right),\;\alpha \neq 1,$$

where α is a real parameter which is known as the entropy-index. One of the most important property of Tsallis entropy is that it has a maximum value determining when each micro-state is equiprobable ($\rho_{j}=1/\Phi$ for all j) and then we get

$$\Upsilon_{\alpha }^{max}=\frac{1-\Phi^{1-\alpha }}{\alpha -1},\;\alpha \neq 1.$$

If α>1 then $\Upsilon_{\alpha }^{max}\to 1/\left(\alpha -1\right)$ and if α→∞ then $\Upsilon_{\alpha }^{max}\to 0$ (see [22]).

Machado [23,24] presented novel formulas for entropy inspired by using the behavior of fractional calculus. The results of the generalized fractional entropy are examined both in usual probability distributions and data series. Moreover, by using the quantum deformed calculus, Hasan et al. [25] introduced a generalized q-entropy.

Numerous issues rule the transmissibility of the infection from the affected to the unaffected. In addition, disease dynamical systems can be investigated at altered rules: the single distinct, small collections of people, and among whole people. Different representations are selected given by the complexity of available data. In their contemporary avatar, computers that generate the numbers and distribution designs of infections simulate systems (see [26,27,28,29]).

The SISE system is formulated with *N* patrons and all the patrons are separated into *n* groups by their joints (junctions) j(j=1,2,…,n). Consequently, it has $N=\sum_{j=1}^{n}N_{j},$ where $N_{j}$ represents the total number of the patron with position j. It is considered that every patron has two positions, the first position is infected (I) and the second position is the susceptible (S). The susceptible patron may be infected with transmission ratio τ, and the infected patron may improve to a susceptible patron with repossession ratio ϱ. Hence, we obtain the equation

$$N_{j}\left(t\right)=S_{j}\left(t\right)+I_{j}\left(t\right)$$

and the discrete system

(1) $$\begin{array}{cc} \; & S_{j}\left(t+1\right)=S_{j}\left(t\right)\left(1-\tau jT\;\;\Upsilon_{\alpha }\left(I_{j}\left(t\right)\right)\right)+\varrho T\;I_{j}\left(t\right)\\ \; & I_{j}\left(t+1\right)=I_{j}\left(t\right)\left(1-\varrho T\;\;\right)+\tau jT\;S_{j}\left(t\right)\;\Upsilon_{\alpha }\left(I_{j}\left(t\right)\right), \end{array}$$

$$\left(0\leq S_{j}\left(0\right)\leq N_{j},\;\;\;0\leq I_{j}\left(0\right)\leq N_{j}\right)$$

where $\Upsilon_{\alpha }\left(I_{j}\left(t\right)\right)$ is the Tsallis entropy introduced by the probability that any given connect points to an infected node and T indicates the time-step measure. It is a value indicating out that scheme (1) is recognized by employing the forward Euler pattern to the continuous SISE system and the equilibrium points (in discrete system they are equal to the fixed points) of structure (1) are similar as for the continuous equivalent. By letting

$$S\left(t\right)=\sum_{j=1}^{n}S_{j}\left(t\right),\;\mathrm{and}\;I\left(t\right)=\sum_{j=1}^{n}I_{j}\left(t\right),$$

system (1) becomes

(2) $$\begin{array}{cc} \; & S\left(t+1\right)=S\left(t\right)\left(1-\tau T\;\;\Upsilon_{\alpha }\left(I\left(t\right)\right)\right)+\varrho T\;I\left(t\right)\\ \; & I\left(t+1\right)=I\left(t\right)\left(1-\varrho T\;\;\right)+\tau T\;S\left(t\right)\;\Upsilon_{\alpha }\left(I\left(t\right)\right), \end{array}$$

$$\left(0\leq S(0)\leq N,\;\;\;0\leq I(0)\leq N,\;t=0,1,2,..\right).$$

Approximate (2) to entropy system, we have

(3) $$\begin{array}{cc} \; & S\left(t+1\right)=S\left(t\right)-\tau T\;\;\Lambda \left(\Upsilon_{\alpha }(I\left(t\right),\Upsilon_{\alpha }(S\left(t\right)\right)+\varrho T\;I\left(t\right)\\ \; & I\left(t+1\right)=I\left(t\right)\left(1-\varrho T\;\;\right)+\tau T\;\Lambda \left(\Upsilon_{\alpha }(I\left(t\right),\Upsilon_{\alpha }(S\left(t\right)\right), \end{array}$$

where $\Lambda \left(\Upsilon_{\alpha }(I\left(t\right),\Upsilon_{\alpha }(S\left(t\right)\right)=\Upsilon_{\alpha }\left(I\left(t\right)\right)\times \Upsilon_{\alpha }\left(S\left(t\right)\right).$

The definition of the function Λ is more general description of the interaction of susceptible and infected individual using entropy. Entropy is a powerful implement for analysis telling the probability distributions of the potential formal of a system, and hence the information encoded in it. Nevertheless, significant information may also be organized in the time-based dynamics, a feature that is not typically taken into account. The notion of scheming entropy based on non-linear designs is utilized to discover spatial structures and processes. Usually, spatial processes have been supposed to be linear, characteristically in relations of a linear auto regressive or heartrending average process. Nevertheless, additional spatial dynamics are possible to display nonlinear types in a technique that is similar to time-based systems. The capacities of nonlinear systems are progressively documented in science, as the restrictions of equilibrium representations in clarifying real-world phenomena convert more seeming. As a consequence, interest is growing in the growth studies in science.

We proceed to conclude the existence of solution of (3).

**Theorem** **1.** *Consider the entropy discrete system of SISE (3). Then it has bounded non-negative solutions if the following hypotheses are achieved*

(4) 0<ϱT<1,α>1.

**Proof.**  By the maximum value of the Tsallis entropy, System (3) implies that

$$\begin{array}{cc} I(1) & =I\left(0\right)\left(1-\varrho T\;\;\right)+\tau T\;\Lambda \left(\Upsilon_{\alpha }(I\left(0\right),\Upsilon_{\alpha }(S\left(0\right)\right)\\ \; & \leq N\left(1\right)+\tau T\Upsilon_{\alpha }^{max}\left(I\right)\times \Upsilon_{\alpha }^{max}\left(S\right)\\ \; & \leq N\left(1\right)+\frac{\tau T}{{\left(\alpha -1\right)}^{2}}. \end{array}$$

By letting α→∞, we have $\lim_{\alpha \to \infty }\frac{\tau T}{{\left(\alpha -1\right)}^{2}}=0$ for all fixed parameters τ and T. Thus, I(1) is bounded by N. Moreover, since $I_{j}\left(0\right)=N_{j}$ with ϱT1 then this yields that for α→∞, the initial solution becomes I(0)=N and consequently the step one of solution becomes I(1)=N(1−ϱT)≥0, which leads to the non-negative solution I. Hence, by induction, one can prove that 0≤I(t)≤N for t=0,1,2,…. By the above construction together with the initial condition S(0)=0, we confirm that S(t)≤N for all t=0,1,2,... Furthermore, since ϱT>0 then S(t)→ϱTI(t)≥0. We indicate that System (3) has a bounded non-negative solution. ☐

## 3. Stability of SISE System

In this section, we aim to study the stability of SISE (1). By substituting $I_{j}\left(t\right)=N_{j}\left(t\right)-S_{j}\left(t\right)$ in the first equation of System (1), we have

(5) $$\begin{array}{cc} \; & S_{j}\left(t+1\right)=S_{j}\left(t\right)\left(1-\tau jT\;\;\Upsilon_{\alpha }\left(I_{j}\left(t\right)\right)\right)+\varrho T\;\left(N_{j}-S_{j}\left(t\right)\right)\\ \; & I_{j}\left(t+1\right)=I_{j}\left(t\right)\left(1-\varrho T\;\;\right)+\tau jT\;S_{j}\left(t\right)\;\Upsilon_{\alpha }\left(I_{j}\left(t\right)\right), \end{array}$$

which is equivalent to the following system

(6) $$\begin{array}{cc} \; & S_{j}\left(t+1\right)=S_{j}\left(t\right)\left(1-\varrho T-\tau jT\;\;\Upsilon_{\alpha }\left(I_{j}\left(t\right)\right)\right)+\varrho T\;N_{j}\left(t\right)\\ \; & I_{j}\left(t+1\right)=I_{j}\left(t\right)\left(1-\varrho T\;\;\right)+\tau jT\;S_{j}\left(t\right)\;\Upsilon_{\alpha }\left(I_{j}\left(t\right)\right). \end{array}$$

The disease free equilibrium of SISE (6) can be computed by the following construction

$$\Xi_{0}\left(S_{1}\left(0\right),\ldots ,S_{n}\left(0\right),I_{1}\left(0\right),\ldots ,I_{n}\left(0\right)\right)=\left(N_{1},\ldots ,N_{n},0,\ldots ,0\right).$$

By employing the linearization matrix method [19] on the system (6) at the point $\Xi_{0}$, we obtain

(7) $$\begin{array}{c} \Psi ={\left(\begin{array}{cc} \psi +\sigma & 0\\ -\psi & \sigma \end{array}\right)}_{2n\times 2n}, \end{array}$$

where ψ (the vector of new infections) and σ (the vector of all other transitions including disease-connected deaths) are non-negative such that ψ+σ is irreducible and Ψ indicates the Jacobi matrix at $\Xi_{0}$ (we assume that this point is unique). Note that

$$\psi ={\left(\begin{array}{ccc} \tau TN_{1}\Upsilon_{\alpha }^{max} & \ldots & \tau TnN_{1}\Upsilon_{\alpha }^{max}\\ & \ldots & \\ \tau TN_{n}n\Upsilon_{\alpha }^{max} & \ldots & \tau TN_{n}n^{2}\Upsilon_{\alpha }^{max} \end{array}\right)}_{n\times n},$$

and

$$\sigma ={\left(\begin{array}{cccc} 1-\varrho T & 0 & \ldots & 0\\ & \ldots & & \\ 0 & \ldots & 0 & 1-\varrho T \end{array}\right)}_{n\times n}.$$

Hence, System (6) approximates to the form

Z(t+1)≈ΨZ(t),Z(t)=(S(t),I(t)),t=0,1,2,...

**Remark** **1.**

- *We used the maximum value of entropy in our system because our suggested system is formulated only for the infected and susceptible persons. We did not include the removed cases R(t) (death and recovery). This variable may be defined by using the maximum entropy $R\left(t\right)=\Upsilon_{\alpha }^{max}\left(I\left(t\right)\right)$ (see [30]).*
- *Note that entropy index is strongly connected to the number of individuals N and the number of groups n, (1≤n≤N), so that when n=N, one would expect the SIS model consequence with non-linear incidence. Very recently, Tsallis and Tirnakli [31] proposed a q-statistical functional arrangement that acts to describe acceptably the existing information for all countries. Reliably, calculations of the dates and altitudes of those peaks in rigorously affected countries become likely unless well-organized actions or vaccines, or functional modifications of the accepted epidemiological approaches, arise.*

### The Basic Reproductive Ratio

The basic reproductive ratio ($\lambda_{0}$) can be explained as the predictable ratio of cases openly produced by one case in a resident where all persons are subject to infection. Mathematically, it is known as the spectral radius of the matrix $\psi {\left(I_{d}-\sigma \right)}^{-1}$ (the largest absolute number of the eigenvalues).

There are other different definitions and formulas can describe the situation properly. This ratio plays an important role to achieve the stability. It has been shown in many studies if $\lambda_{0}>1$ then we indicate an unstable situation and if $\lambda_{0}<1$ then the situation is asymptotically stable, while the case $\lambda_{0}=1$ indicates the stability, but not being asymptotic [19]. Recently, for COVID-19, researchers suggested the case fatality rate (CFR, the aim is to reduce this ratio) [32]

$$CFR\left(t\right)=\frac{D(t)}{I(t)},\;t=0,1,2,..\;,$$

where *D* indicates the number of dying people. For example, if the number D=10 and I=500, then the ratio is 2%. Simultaneously, if it is recorded that there are 500 susceptible persons then

$$CFR_{N}\left(t\right)=\frac{D(t)}{I(t)+S(t)}=\frac{10}{1000}=1\%.$$

The aim is to reduce the rate $\lambda_{0}$ or CFR by isolated position and keep cleaning the environment of the person. In our discussion, we suggest to involve the entropy evaluation for this rate. In [33] the authors formulated $\lambda_{0}$ by using the probability of the survival function *P* as follows (for discrete data):

$$\lambda_{0}=\frac{I(t)\times P(t)}{N(t)},\;t=0,1,2,..\;N\left(t\right)=I\left(t\right)+S\left(t\right).$$

From the above example I=500,D=10 and N=1000, we have

$$\lambda_{0}=\frac{500\times 0.98}{1000}=49\%.$$

The idea of the probability of the survival function is not suitable for COVID-19. Therefore, based on our SISE system, we suggest to use the maximum entropy $\Upsilon_{\alpha }^{max}\left(I\right)$ as follows:

$${\left(\lambda_{0}\right)}_{\alpha }=\frac{I\left(t\right)\times \Upsilon_{\alpha }^{max}\left(I\left(t\right)\right)}{N(t)},\;t=0,1,2,\ldots \;\alpha \neq 1.$$

Based on the above data, we indicate the following results ${\left(\lambda_{0}\right)}_{2}=0.5\approx \lambda_{0},$ that is $\lim_{\alpha \to 2}{\left(\lambda_{0}\right)}_{\alpha }=\lambda_{0}.$ Note that $,\;{\left(\lambda_{0}\right)}_{3}=0.25,{\left(\lambda_{0}\right)}_{4}=0.16,\ldots$. We conclude that the ratio decreases whenever α increases. Hence, the SISE system is stable, while for α<1, the system is unstable. For example, when α=0.5 and the probability P(I)=0.1 this implies that $\Phi =\frac{1}{P(I)}=10,$ we get $\Upsilon_{\alpha }^{max}(I\left(t\right)=\frac{1-10^{0.5}}{-0.5}=4.324;$ which leads ${\left(\lambda_{0}\right)}_{0.5}=4.324\times 0.5=2.162>1.$

From above, we conclude that Theorem 1 can be extended to include the stability as follows:

**Theorem** **2.** *Consider the entropy discreet system of SISE (3). If the conditions*

(8) 0<ϱT<1,α>1

*hold. Then every solution of (3) is bounded non-negative and stable satisfying the basic reproductive ratio*

(9) $${\left(\lambda_{0}\right)}_{\alpha }=\frac{I\left(t\right)\times \Upsilon_{\alpha }^{max}\left(I\left(t\right)\right)}{N(t)},\;t=0,1,2,...\;.$$

The survival function (or it called reliability function) is a function that offers the probability that a patient, scheme, or other thing of concern will survive further than any indicated time and it is one of the techniques to define and show survival data. It states as the probability that a subject survives longer than time t. The distribution of survival times may be approximated well by a function such as the exponential distribution. Numerous distributions are usually utilized in survival analysis, containing the exponential, Tsallis entropy, gamma, normal and log-logistic. These distributions are formulated by parameters. The entropy optimization principle (includes the maximum entropy) converts it from a measure of information into an implement of statistics conclusively [34]. Since the higher maximum entropy goes to Tsallis entropy (see [35]), then it is a confidence to employ this fact to define CFR.

## 4. Applications

In this section, we use live data to examine our theoretical results, especially the stability of the SISE system by using ${\left(\lambda \right)}_{\alpha }.$ Table 1 shows the data from the first infected countries until the end of May. The rate of death is given by using CFR ${}_{N}\left(t\right)$. The basic reproduction ratio is evaluated by using ${\left(\lambda_{0}\right)}_{\alpha }$ for some α>1. We use the conditions of Theorem 2 to get non-negative bounded and stable solution. One can consider the following system

**Example** **1.** *Consider the following system*

(10) $$\begin{array}{cc} \; & S_{j}\left(t+1\right)=aS_{j}\left(t\right)(1-S_{j}\left(t\right)\times \Upsilon_{\alpha }^{max}\left(I\right)\\ \; & I_{j}\left(t+1\right)=bI_{j}\left(t\right)\left(1-I_{j}\left(t\right)\right)\times \Upsilon_{\alpha }^{max}\left(I\right), \end{array}$$

**Figure 1:**
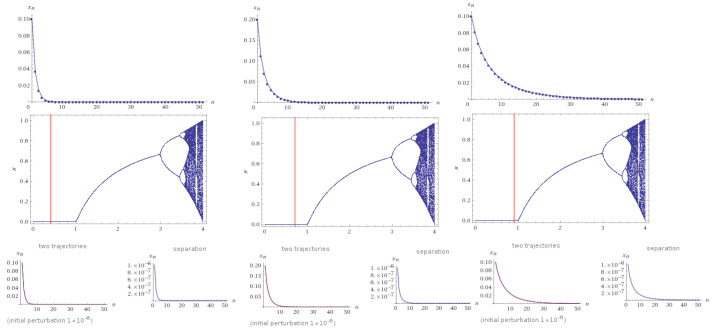
Bifurcation diagrams of System (10) with 100 iterations, when a=b=0.4 and α=2 (**left** column, with initial condition $S_{0}=I_{0}=0.1$). The **middle** column is for a=b=0.7 and initial condition $S_{0}=I_{0}=0.2$ The **right** column indicates the case a=b=0.9 under the initial condition $S_{0}=I_{0}=0.1.$ All cases indicate a stable limit cycle of period one. The red line indicates the values of each case.

*where a:=τT,b:=ϱT. Let a=b=0.4, initial condition $\left(S_{0},I_{0}\right)=\left(0.1,0.1\right)$ and α=2, we have a stable limit cycle for the system of period one (see Figure 1). The red line shows the values of each considered case.*

**Example** **2.** *Consider the following system*

(11) $$\begin{array}{cc} \; & S_{j}\left(t+1\right)=-aS_{j}{\left(t\right)}^{2}\times \Upsilon_{\alpha }^{max}\left(I\right)+I_{j}\left(t\right)+1\\ \; & I_{j}\left(t+1\right)=bS_{j}\left(t\right)\times \Upsilon_{\alpha }^{max}\left(I\right), \end{array}$$

*where a:=τT,b=ϱT and α=2 with (0.2,0.6) as initial point. In all figures, the red line indicates the values of each considered case as follows:*

**Figure 2:**
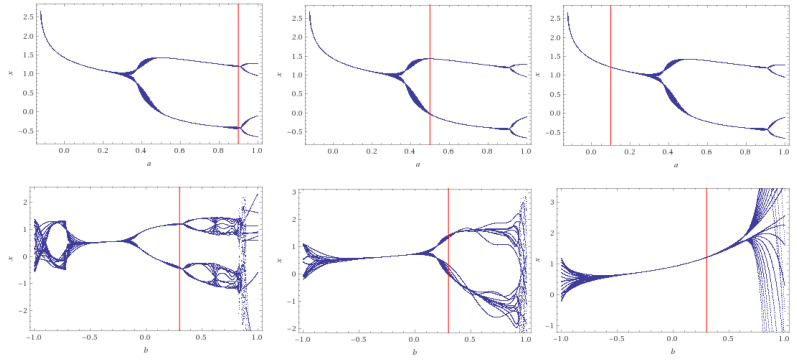
Bifurcation diagrams of System (11), when a=0.9,b=0.3 (**left**), a=0.5,b=0.3 (**middle**) and a=0.1,b=0.3 (**right**). The value of the α=2. Note that x=S(t).

**Figure 3:**
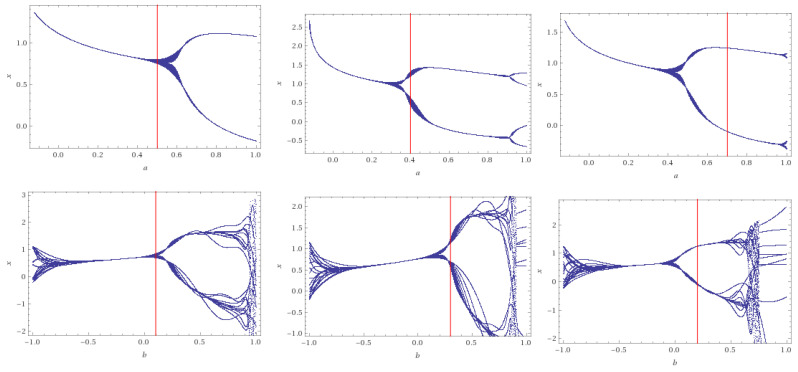
Bifurcation diagrams of System (11), when a=0.1,b=0.5 (**left**), a=0.4,b=0.3 (**middle**, with a limit cycle of period 2) and a=0.7,b=0.2 (**right**, with a limit cycle of period 2). The red line represents the values of each considered case.

- *For a=0.9,b=0.3, the system has a limit cycle with period 4, while for a=0.5,b=0.3 the system has a limit cycle with period 2.*
- *For a=0.1,b=0.3, it has no limit cycle (see Figure 2). The positive fixed point of the third case is $\varphi =0.3={\left(\lambda \right)}_{2}$ in the USA’s situation. While there are two positive fixed points (equilibrium point in the difference equation) for the first case, $\varphi_{1}=0.7={\left(\lambda \right)}_{2}$ for Spain and $\varphi_{2}={\left(\lambda \right)}_{3}$ for Russia.*
- *Also, for the initial condition =(0,0) and the case a=0.9,b=0.5, we get two positive fixed points $\varphi_{1}=0.8$ and $\varphi_{2}=0.4={\left(\lambda \right)}_{2}$ for Russia.*
- *Figure 3 represents to the bifurcation behavior of the system (9) with the initial condition (0,0),a=0.1,b=0.5 (left), a=0.4,b=0.3 (middle) and a=0.1,b=0.5 (right). In the second case, we have a limit cycle of period 2 and two positive fixed points $\varphi_{1}=0.9$ and $\varphi_{2}=0.27={\left(\lambda \right)}_{3}$ for Brazil.*
- *The last case a=0.7,b=0.2, we have two positive fixed points $\varphi_{1}=0.7={\left(\lambda \right)}_{2}$ (Spain) and $\varphi_{2}=0.16={\left(\lambda \right)}_{4}$ (Brazil).*

Note that the simplest situation is the case where there is no recuperation rate. This leads to an SI-like model, so that the pathogen infects all individuals on the long run. The simple continuous SI model has the logistic function as a solution and its discretized version is the logistic map, which presents the traditional bifurcation diagram as stable solutions.

## 5. Conclusions

The correct balance between short- and long-term data loading in the world of big data has the following strategies:

- An applied perception based on the main usages for the data;
- The data constructions wanted for analysis;
- The relevancy of the data over time;
- Development with little organization is essential.

We prepared a new formula of the basic reproductive ratio ${\left(\lambda_{0}\right)}_{\alpha },$ which is defined by the Tsallis entropy. The formula is useful for the stability of SISE system involving the Tsallis entropy. It is related to long time data (by taking in account the above stratifies, it may modify for short time data). We applied the suggested system by using live data regarding COVID-19.

## Figures and Tables

**Table 1:** Data of COVID-19 until end of May (α=2,3,4).

Country Name	Total (N)	Infected Number (*I*)	Death	CFR ${}_{N}$	${\left(\lambda \right)}_{2}$	${\left(\lambda \right)}_{3}$	${\left(\lambda \right)}_{4}$
USA	1,837,170	599,867	106,195	15%	0.326	0.163	0.097
Brazil	514,992	279,096	29,341	12%	0.541	0.275	0.162
Russia	405,843	171,883	4693	1%	0.423	0.211	0.127
Spain	286,509	196,958	27,127	12%	0.687	0.343	0.206

## References

[1] Nicolas B. (2011). A Short History of Mathematical Population Dynamics.

[2] Kermack W.O., McKendrick A.G. (1927). A contribution to the mathematical theory of epidemics. Proc. R. Soc. A.

[3] Chang Z., Meng X., Zhang T. (2019). A new way of investigating the asymptotic behaviour of a stochastic sis system with multiplicative noise. Appl. Math. Lett..

[4] Huang W., Han M., Liu K. (2017). Dynamics of an SIS reaction-diffusion epidemic model for disease transmission. Math. Biosci. Eng..

[5] Liu M., Chang Y., Wang H., Li B. (2018). Dynamics of the impact of twitter with time delay on the spread of infectious diseases. Int. J. Biomath..

[6] Newman M.E.J. (2003). The structure and function of complex networks. SIAM Rev..

[7] Pastor-Satorras R., Vespignani A. (2001). Epidemic spreading in scale-free networks. Phys. Rev. Lett..

[8] Pastor-Satorras R., Vespignani A. (2002). Immunization of complex networks. Phys. Rev. E.

[9] Zhou T., Liu J., Bai W., Chen G., Wang B.H. (2006). Behaviors of susceptible-infected epidemics on scale-free networks with identical infectivity. Phys. Rev. E.

[10] Wu Q., Fu X. (2016). Immunization and epidemic threshold of an SIS model in complex networks. Physica A.

[11] Allen L.J. (1994). Some discrete-time SI, SIR, and sis epidemic models. Math. Biosci..

[12] Liu J., Peng B., Zhang T. (2015). Effect of discretization on dynamical behavior of SIR and sir models with nonlinear incidence. Appl. Math. Lett..

[13] Hu Z., Teng Z., Zhang L. (2014). Stability and bifurcation analysis in a discrete sir epidemic model. Math. Comput. Simul..

[14] Elaydi S. (2005). An Introduction to Difference Equations.

[15] Mickens R.E. (1999). Discretizations of nonlinear differential equations using explicit nonstandard methods. J. Comput. Appl. Math..

[16] Jang S., Elaydi S. (2003). Difference equations from discretization of a continuous epidemic model with immigration of infectives. Can. Appl. Math. Q..

[17] Li L., Sun G., Jin Z. (2010). Bifurcation and chaos in an epidemic model with nonlinear incidence rates. Appl. Math. Comput..

[18] Castillo-Chavez C., Abdul-Aziz Y. (2002). Discrete-time SIS models with simple and complex population dynamics. Inst. Math. Appl..

[19] Allen J.S.L., van den Driessche P. (2008). The basic reproduction number in some discrete-time epidemic models. J. Diff. Eq. Appl..

[20] Wang X., Wang Z., Shen H. (2019). Dynamical analysis of a discrete-time SIS epidemic model on complex networks. Appl. Math. Lett..

[21] Tsallis C. (1988). Possible generalization of Boltzmann-Gibbs statistics. J. Stat. Phys..

[22] Ramírez-Reyes A., Hernández-Montoya A.R., Herrera-Corral G., Domínguez-Jiménez I. (2016). Determining the entropic index q of Tsallis entropy in images through redundancy. Entropy.

[23] Jose Tenreiro M. (2014). Fractional order generalized information. Entropy.

[24] Jose Tenreiro M. (2019). Fractional Renyi entropy. Eur. Phys. J. Plus.

[25] Hasan A.M., AL-Jawad M.M., Jalab H.A., Shaiba H., Ibrahim R.W., AL-Shamasneh A.A.R. (2020). Classification of Covid-19 Coronavirus, Pneumonia and Healthy Lungs in CT Scans Using Q-Deformed Entropy and Deep Learning Features. Entropy.

[26] Ibrahim R.W. (2013). The fractional differential polynomial neural network for approximation of functions. Entropy.

[27] Ibrahim R.W. (2020). Utility function for intelligent access web selection using the normalized fuzzy fractional entropy. Soft Comput..

[28] Jalab H.A., Subramaniam T., Ibrahim R.W., Kahtan H., Noor N.F.M. (2019). New Texture Descriptor Based on Modified Fractional Entropy for Digital Image Splicing Forgery Detection. Entropy.

[29] Ibrahim R.W., Maslina D. (2018). Analytic study of complex fractional Tsallis’ entropy with applications in CNNs. Entropy.

[30] Yong T. (2020). Maximum entropy method for estimating the reproduction number: An investigation for COVID-19 in China. medRxiv.

[31] Tsallis C., Tirnakli U. (2020). Predicting COVID-19 peaks around the world. Front. Phys..

[32] Pennings P., Yitbarek S., Ogbunu B. COVID19 in Numbers-R_0_, the Case Fatality Rate and Why We Need to Flatten the curve.webm. https://en.wikipedia.org/wiki/File:COVID19_in_numbers-_R0,_the_case_fatality_rate_and_why_we_need_to_flatten_the_curve.webm.

[33] Heffernan J.M., Smith R.J., Wahl L.M. (2005). Perspectives on the basic reproduction ratio. J. R. Soc. Interface.

[34] He D., Huang Q., Gao J. (2012). A new entropy optimization model for graduation of data in survival analysis. Entropy.

[35] Singh V.P., Sivakumar B., Cui H. (2017). Tsallis entropy theory for modeling in water engineering: A review. Entropy.

